# Glucokinase Regulatory Protein Genetic Variant Interacts with Omega-3 PUFA to Influence Insulin Resistance and Inflammation in Metabolic Syndrome

**DOI:** 10.1371/journal.pone.0020555

**Published:** 2011-06-06

**Authors:** Pablo Perez-Martinez, Javier Delgado-Lista, Antonio Garcia-Rios, Jolene Mc Monagle, Hanne L. Gulseth, Jose M. Ordovas, Danielle I. Shaw, Brita Karlström, Beata Kiec-Wilk, Ellen E. Blaak, Olfa Helal, Małgorzata Malczewska-Malec, Catherine Defoort, Ulf Risérus, Wim H. M. Saris, Julie A. Lovegrove, Christian A. Drevon, Helen M. Roche, Jose Lopez-Miranda

**Affiliations:** 1 Lipid and Atherosclerosis Unit, IMIBIC/Reina Sofia University Hospital/University of Cordoba, and CIBER Fisiopatologia Obesidad y Nutricion (CIBEROBN), Instituto de Salud Carlos III, Cordoba, Spain; 2 Nutrigenomics Research Group, UCD School of Public Health and Population Science, UCD Conway Institute, University College Dublin, Dublin, Ireland; 3 Department of Endocrinology, Oslo University Hospital, Oslo, Norway; 4 Nutrition and Genomics Laboratory, JM-USDA Human Nutrition Research Center on Aging at Tufts University, Boston, Massachusetts, United States of America; 5 Department of Epidemiology and Population Genetics, Centro Nacional Investigación Cardiovasculares (CNIC), Madrid, Spain; 6 Department of Food and Nutritional Sciences and the Institute for Cardiovascular and Metabolic Research (ICMR), University of Reading, Reading, Berkshire, United Kingdom; 7 Clinical Nutrition and Metabolism, Department of Public Health and Caring Sciences, Uppsala University, Uppsala, Sweden; 8 Department of Clinical Biochemistry, and Department of Metabolic Diseases, Jagiellonian University Medical College, Krakow, Poland; 9 Department of Human Biology, NUTRIM, School for Nutrition, Toxicology and Metabolism, Maastricht, The Netherlands; 10 INSERM 476, Lipid nutrients and prevention of metabolic diseases, INRA, 1260, Université de la Méditerranée, Marseille, France; 11 Department of Nutrition, Institute of Basic Medical Sciences, Faculty of Medicine, University of Oslo, Oslo, Norway; University of Leuven, Rega Institute, Belgium

## Abstract

Glucokinase Regulatory Protein (GCKR) plays a central role regulating both hepatic triglyceride and glucose metabolism. Fatty acids are key metabolic regulators, which interact with genetic factors and influence glucose metabolism and other metabolic traits. Omega-3 polyunsaturated fatty acids (n-3 PUFA) have been of considerable interest, due to their potential to reduce metabolic syndrome (MetS) risk.

**Objective:**

To examine whether genetic variability at the *GCKR* gene locus was associated with the degree of insulin resistance, plasma concentrations of C-reactive protein (CRP) and n-3 PUFA in MetS subjects.

**Design:**

Homeostasis model assessment of insulin resistance (HOMA-IR), HOMA-B, plasma concentrations of C-peptide, CRP, fatty acid composition and the *GCKR* rs1260326-P446L polymorphism, were determined in a cross-sectional analysis of 379 subjects with MetS participating in the LIPGENE dietary cohort.

**Results:**

Among subjects with n-3 PUFA levels below the population median, carriers of the common C/C genotype had higher plasma concentrations of fasting insulin (P = 0.019), C-peptide (P = 0.004), HOMA-IR (P = 0.008) and CRP (P = 0.032) as compared with subjects carrying the minor T-allele (Leu446). In contrast, homozygous C/C carriers with n-3 PUFA levels above the median showed lower plasma concentrations of fasting insulin, peptide C, HOMA-IR and CRP, as compared with individuals with the T-allele.

**Conclusions:**

We have demonstrated a significant interaction between the *GCKR* rs1260326-P446L polymorphism and plasma n-3 PUFA levels modulating insulin resistance and inflammatory markers in MetS subjects. Further studies are needed to confirm this gene-diet interaction in the general population and whether targeted dietary recommendations can prevent MetS in genetically susceptible individuals.

**Trial Registration:**

ClinicalTrials.gov NCT00429195

## Introduction

The metabolic syndrome (MetS) represents a combination of cardio-metabolic risk factors, including central obesity, insulin resistance, hypertension, and dyslipidemia [1]. Low-grade inflammation is characteristic of MetS and numerous studies have now confirmed that plasma concentrations of C-reactive protein (CRP), the most commonly measured biomarker of inflammation, are elevated in individuals with MetS [2]. The expression of the MetS is probably due to complex interactions between genetic and environmental factors. Moreover, evidence suggest that some people are genetically predisposed to insulin resistance [3], which may represent an underlying mechanism for these metabolic alterations. Among the environmental factors, diet may be the most directly involved in the genetic modulation of the different intermediate and final phenotypes of diabetes mellitus (T2DM) and cardiovascular disease. Omega-3 polyunsaturated fatty acids (n-3 PUFA) are specially relevant, due to their potential ability to lower risk of coronary heart disease and MetS [4], [5], [6]. However, limited and inconsistent data are available concerning the relation between n-3 PUFA and glucose metabolism. Furthermore, data from a recent prospective study including 36,328 women in the Women's Health Study, suggest an increased risk of T2DM, especially with higher intakes of n-3 PUFA [7]. This study supports the notion that general recommendations may not be benefit equally to all individuals underscoring the need to elucidate how certain dietary components may modulate the risk conferred by genetic susceptibility due to variation in genes involved in the etiology of MetS.

In the liver and pancreatic islet cells, glucokinase regulatory protein (GCKR) modifies glucokinase, which functions as a glucose sensor responsible for glucose phosphorylation in the first step of glycolysis. In a murine experimental model, adenoviral-mediated overexpression of GCKR in liver increased glucokinase activity, which led to lowered blood glucose and increased triglycerides concentrations [8]. Along these lines we have provided evidence that common variation at the *GCKR* locus, more specifically the functional rs1260326 (Pro446Leu) SNP, was associated with opposite effects on fasting plasma triglycerides and glucose concentrations in several populations [9]. Moreover Beer et al. demonstrated that GCKR T-allele Pro446Leu has reduced regulation by physiological concentrations of phosphate esters fructose 6 (F6P), resulting indirectly in increased GCK activity [10]. Altered GCK regulation in liver is predicted to enhance glycolytic flux, promoting hepatic glucose metabolism and elevating concentrations of malonyl-CoA, a substrate for de novo lipogenesis, providing a mutational mechanism for the reported association of this variant with raised triglycerides and lower glucose levels [10]. Taken together, these data support the central position of GCKR in pathways regulating hepatic triglyceride as well as glucose metabolism in humans. In addition, we confirmed recent findings that the same SNP is consistently associated with CRP levels [9]. In view of the physiological role of GCKR in glucose homeostasis and the link between insulin resistance, inflammation and dietary fat, our primary aim was to examine whether genetic variability at the GCKR gene locus in MetS subjects might be associated with variability in insulin response and CRP levels to n-3 PUFA. Moreover we also explored other potential gene-nutrient interaction, such as saturated fatty acids (SFA), monounsaturated fatty acids (MUFA) and n-6 PUFA.

## Results

Baseline demographic and biochemical characteristics according to the *GCKR* rs1260326-P446L polymorphism are presented in **Table 1**. Genotype distributions did not deviate from Hardy-Weinberg expectations. Given the low genotype frequencies of individuals homozygous for the minor alleles, and because the analysis did not suggest a recessive mode of inheritance, we analyzed the rs1260326 SNP using two genotype categories. The number of men and women enrolled in this study was 174 (55 C/C and 119 C/T+T/T) and 205 (70 C/C and 135 C/T+T/T), respectively. Consistent with previous studies, the variant *GCKR* T-allele Pro446Leu (C/T+T/T) displayed higher plasma levels of triglycerides and CRP but lower fasting glucose concentrations as compared to homozygous Pro446 carriers (C/C) (**Table 1**). No other significant baseline differences were observed in relation to age, BMI, fasting plasma lipids, and insulin concentration by genotype.

**Table 1:** Characteristics of participants at the baseline according to the *GCKR* rs1260326-P446L polymorphism.

	rs1260326		
	C/C	C/T+T/T	* *P value*
n	125	274	
Age, years	55.02 (0.8)	53.91 (0.5)	0.259
BMI, kg/m2	32.59(0.4)	32.54 (0.2)	0.927
Total cholesterol, mmol/L	5.25 (0.07)	5.34 (0.06)	0.358
LDL-C, mmol/L	3.09 (0.10)	3.29 (0.06)	0.095
HDL-C, mmol/L	1.12 (0.01)	1.09 (0.01)	0.261
TG, mmol/L	1.62 (0.06)	1.84 (0.05)	**0.016**
ApoB, g/L	1.00 (0.01)	1.02 (0.01)	0.420
ApoA-1, g/L	1.39 (0.02)	1.4 (0.01)	0.657
Glucose (mmol/L)	6.09 (0.06)	5.87 (0.05)	**0.012**
Insulin (mU/L)	9.97 (0.52)	9.97 (0.33)	0.997
CRP (mg/L)	4.39 (0.31)	5.52 (0.28)	**0.016**

*P<0.05.

**Figure 1:**
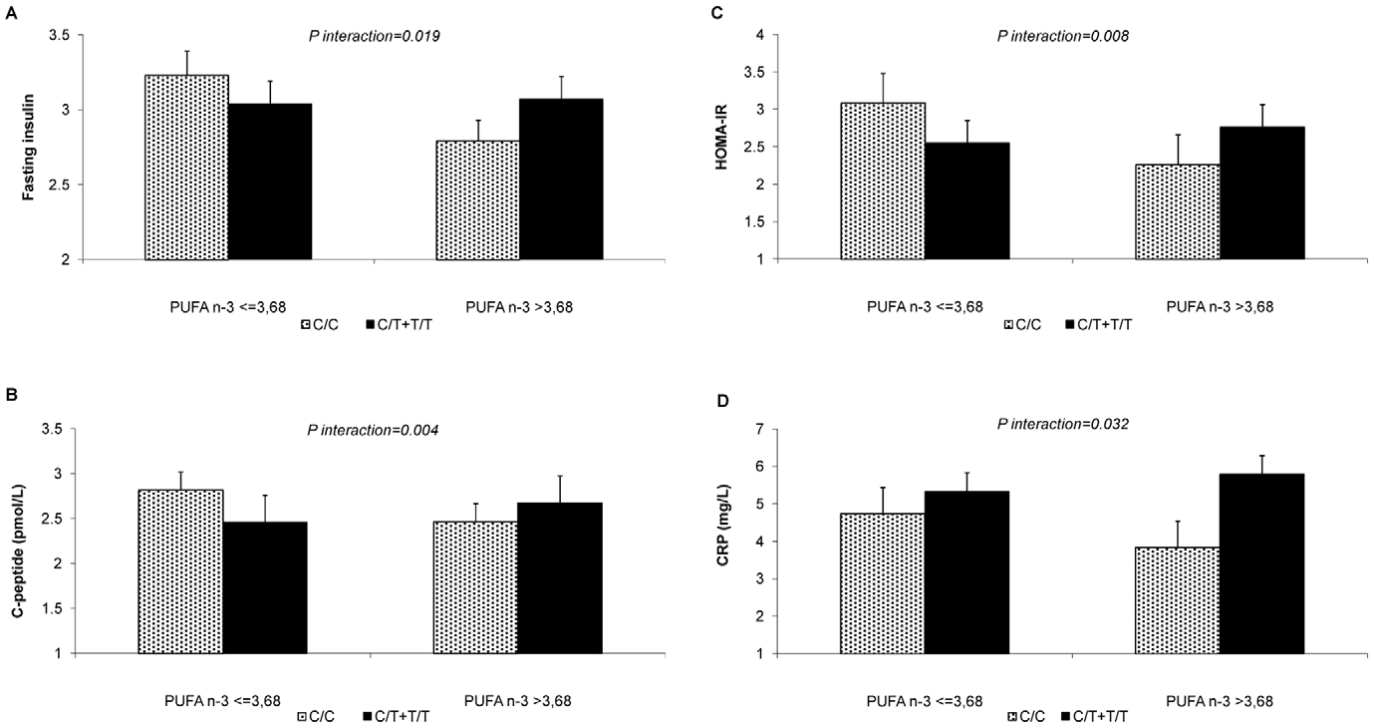
Interaction between the *GCKR* rs1260326-P446L polymorphism and plasma concentrations of omega-3 polyunsaturated fatty acids (n-3 PUFA), above or below the median within the same genotype group of fasting insulin (A), C-peptide levels (B), homeostasis model assessment of insulin resistance (HOMA-IR) (C) and C-reactive protein levels (CRP) (D). Values are means ± SD. P values were adjusted for age, sex, BMI, anti-hypertension pharmacological treatment and LIPGENE centre of origin.

Given that the aim of this study was to investigate potential gene-nutrient interactions, we examined the associated effect of the *GCKR* rs1260326-P446L polymorphism on glucose metabolism according to plasma fatty acid status. Gene-nutrient interactions between the rs1260326 SNP and plasma level of n-3 PUFA were found. In the whole cohort, this SNP interacted with plasma n-3 PUFA levels, which were significantly associated with insulin resistance. Among subjects with n-3 PUFA levels below the median (≤3.68), the C/C genotype was associated with higher fasting insulin concentrations (P = 0.019), C-peptide (P = 0.004) and HOMA-IR (P = 0.008) as compared to subjects carrying the minor T-allele (**Figure 1**). In contrast, C/C homozygotes with n-3 PUFA levels above the median (>3.68) showed lower plasma concentration of fasting insulin, C-peptide, and HOMA-IR, compared with individuals carrying the T-allele (**Figure 1**). HOMA-B did not differ between participants with different genotypes. On the other hand we also measured the serum CRP levels and the effect of the *GCKR* rs1260326-P446L polymorphism according to plasma fatty acid status. With the highest level of n-3 PUFA, C/C subjects also showed lower CRP levels compared with subjects carrying the T-allele (P = 0.032) (**Figure 1D**). We also examined the effect of this polymorphism on plasma triglycerides concentrations, the main phenotype associated with GCKR, and with plasma adiponectin levels. No significant differences were observed for these both parameters related to the plasma level of n-3 PUFA. Furthermore there were no significant interactions between other groups of plasma fatty acids (SFA, MUFA, n-6 PUFA, n-6/n-3 and the n-3/n-6 PUFA ratios), and GCKR rs1260326-P446L polymorphism on glucose metabolism, triglycerides, CRP and adiponectin plasma levels.

**Figure 2:**
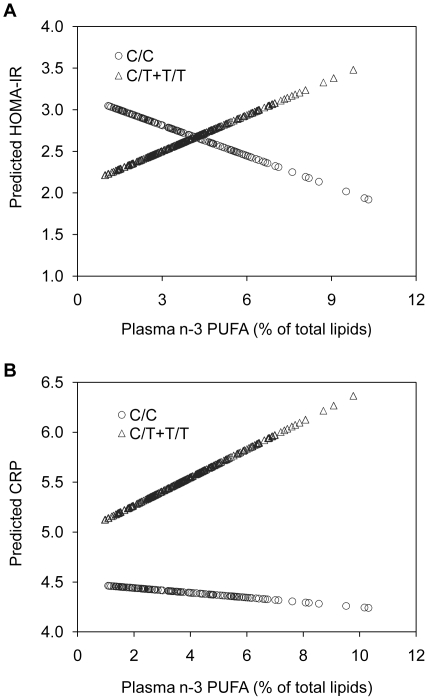
Predicted values for homeostasis model assessment of insulin resistance (HOMA-IR) (A) and C-reactive protein levels (CRP) (B) for the *GCKR* rs1260326-P446L polymorphism. A difference was observed between the genotype groups, with the minor T-allele genotype group (triangles) appearing to be “high responders” to plasma concentration of omega-3 polyunsaturated fatty acids (n-3 PUFA) and the major C allele homozygote group (circles) appearing to be “low responders”.

Finally, a linear regression model including the original covariates was applied to create predicted values of HOMA-IR and CRP levels according to genotype at the rs1260326 polymorphism (**Figure 2**). The genotype groups exhibit striking differences in the predicted changes in HOMA-IR and CRP in relation to plasma n-3 PUFA concentrations. Thus, from baseline data, the model predicts that in individuals carrying the minor T-allele for rs1260326, an increase in plasma n-3 PUFA would elicit a considerable raise in HOMA-IR and CRP levels. This increase would not be seen in the major C-allele homozygote.

## Discussion

Our findings support the hypothesis that the *GCKR* rs1260326-P446L polymorphism influences insulin resistance by interacting with plasma n-3 PUFA levels in MetS patients. Thus, among subjects with low plasma n-3 PUFA levels, the C/C genotype was associated with higher fasting insulin concentrations, C-peptide levels and HOMA-IR compared to subjects carrying the minor T-allele. In contrast, C/C subjects with the highest level of plasma n-3 PUFA, showed lower fasting insulin concentrations, C-peptide levels and HOMA-IR, compared to subjects with the T-allele. Moreover, they also showed lower CRP levels. These novel associations and gene-nutrient interactions might be important for improving therapeutic efficacy of dietary recommendations with a ‘personalised nutrition’ approach in MetS patients.

Common variants in *GCKR*, most often the minor T-allele of the rs780094 SNP (or rs1260326, a variant in high linkage disequilibrium), have been reported by multiple candidate gene and GWAS to be associated with decreased fasting glucose [9], [11], [12], [13], [14], increased serum triglyceride [9], [11], [15], [16] and CRP levels [9], [17], [18]. However, many published claims of gene-biomarker and gene-disease associations have not been replicated when studied in independent populations. In this regard, the present study replicates previous associations of the *GCKR* rs1260326-P446L polymorphism with metabolic traits, including fasting glucose, TAG and CRP levels. Moreover, diet has been considered important in the modulation of metabolic traits related to the MetS. In the last decade more attention has been paid to the impact of the quality of dietary fat, independent of total amount. Among dietary components, n-3 PUFA have been shown to confer some cardiovascular benefits [5], [6], although limited and inconsistent data have been reported on the effects of n-3 PUFA on glucose metabolism. Thus, data from the Nurses' Health studies suggested an increased risk of T2DM with high consumption of n-3 PUFA [19]. These results were consistent with recent findings from the Women's Health Study of which higher but not lower amounts of n-3 PUFA consumption were associated with an increased risk of diabetes [7]. In contrast, data from other studies like the ARIC study [20], and the Kuopio Ischemic Heart Disease Risk factor study [21], showed no association between omega-3 fatty acids and diabetes risk. The question arises whether gene-nutrient interactions such as *GCKR* and n-3 PUFA, together with other confounding factors, might be at the root of such inconsistencies. Our data show that subjects with the less common T-allele and with plasma n-3 PUFA levels above the median, showed higher plasma concentrations of fasting insulin, C-peptide and HOMA-IR, as compared to C/C subjects, suggesting that some individuals may be more resistant (hypo-responders) to dietary changes than others [22], [23]. Thus, general recommendations (i.e. high intake of n-3 PUFA) may not be equally benefit all individuals.

Our study is, to our knowledge, the first to report interactions between the *GCKR* locus, plasma fatty acid and glucose metabolism in subjects with the MetS. The *GCKR* rs1260326-P446L SNP interacted with plasma n-3 PUFA to influence insulin resistance, suggesting the potential sensitivity of this SNP to dietary factors. In contrast, no differences were observed with other types of fatty acids in plasma such as MUFA, n-6 PUFA or SFA. Genetic as well as functional data indicate that GCKR plays a key role in glucose metabolism and intermediate phenotypes. Although we did not perform functional studies, data from Vaxillaire *et al.* suggest that the lower fasting blood glucose in the T-allele carriers is probably due to improved hepatic glucose disposal and insulin sensitivity; this may be due to a direct effect on the hepatic glucose metabolism, as GCKR closely interacts with GCK in the hepatocytes, depending on glucose concentrations, to regulate the amount and activity of GCK and, consequently, the hepatic glucose disposal [12]. As previously shown we observed a gene-fatty acid interaction between the rs1260326 SNP and the levels of n-3 PUFA in plasma. PUFAs are known regulators of hepatic gene transcription, exerting their effects on transcription factors such as PPARα, SREBP-1c, or HNF-4α. Fatty acid-mediated regulation of hepatic genes by HNF-4α has been implicated in the regulation of carbohydrate metabolism through GCK, phosphoenolpyruvate carboxykinase and L-pyruvate kinase [24]. Thus, the decrease in both GCK (a key enzyme of glycolysis) and G6PDH (the rate-limiting enzyme of the pentose phosphate pathway) activities, leading to a reduction in two key metabolites of glucose metabolism (G6P and X5P), favors a PUFA-mediated inhibition of ChREBP translocation. Consistent with the observation that GCK gene expression is inhibited by PUFAs [25], recent evidence shows that only PUFAs, but not SFA or MUFA, inhibit GCK activity by reducing the amount of total GCK protein content. Based on these results we may speculate that n-3 PUFA fatty acids interact with the *GCKR* gene to influence insulin resistance and this is particularly noteworthy as insulin resistance is believed to be an important factor linking metabolic abnormalities in patients with the MetS.

Moreover, our findings suggest that this gene-nutrient interaction also modifies CRP levels. On the basis of the current evidence MetS is associated with low-grade chronic inflammation characterized by inflamed adipose tissue with increased macrophage infiltration. This inflammation may link obesity and insulin resistance. We have observed that subjects carrying the C/C genotype and higher level of plasma n-3 PUFA had a lower grade of inflammation as measured by serum CRP level. This novel gene-nutrient interaction may be cautiously interpreted as a protection against chronic inflammation associated to the MetS. The LIPGENE cohort is a carefully characterized population, and the multicentre origin of the patients allows extrapolation of the results to the European population. However, LIPGENE design is cross sectional, preventing to prove causality.

In conclusion, our results support the notion that n-3 PUFA may play a contributing role in triggering insulin resistance and serum levels of CRP by interacting with a genetic variant at *GCKR* gene locus. Based on these data, and on the observed genotype-dependent responses, we can infer that a recommendation to increase n-3 PUFA could have an augmented beneficial effect on insulin resistance and inflammatory markers only among MetS patients carrying the C/C genotype, which might in turn have implications with respect to cardiovascular risk.

## Materials and Methods

The detailed design of the present study has been described in the recent articles from the LIPGENE cohort [22], [26], [27] and summarized below.

### Ethics Statement

The study was performed in accordance with the Helsinki Declaration of 1975 as revised in 1983, and was approved by the local ethics committees at each centre (Research Ethics Committee of the Adelaide and Meath Hospital and St. James' Hospital, Dublin, Ireland; Berkshire Local Research Ethics Committee and University of Reading Research Ethics Committee, UK; the Norwegian Data Inspectorate and by the Regional Committee for Ethics in Medical Research, Oslo, Norway; Comité Consultatif De Protection Des Personnes Dans La Recherche Biomédicale – MARSEILLE 1, France; the Medical Ethical Committee of Maastricht University/University Hospital Maastricht, The Netherlands; the Bioethical Committee of the Jagiellonian University, Medical College Krakow, Poland; Regional Ethical Review Board Uppsala, Sweden; and Comité Ético de Investigación Clínica, Hospital Universitario Reina Sofía, Cordoba, Spain). All subjects provided written informed consent before any study related procedure. The study was registered with the US National Library of Medicine Clinical Trials registry (NCT00429195).

### Subjects

The participants, aged 35–70 years and BMI 20–40 kg/m^2^, were recruited for the LIPGENE dietary intervention study from eight European countries (Ireland, UK, Norway, France, The Netherlands, Spain, Poland and Sweden). Subject eligibility was determined using a modified version of the NCEP criteria for the MetS [28], where subjects were required to fulfil at least three of the following five criteria: waist circumference >102 cm (men) or >88 cm (women); fasting plasma concentration of glucose 5.5–7.0 mmol/L; TAG≥1.5 mmol/L; HDL cholesterol <1.0 mmol/L (men) or <1.3 mmol/L (women); and blood pressure ≥130/85 mmHg, or treatment of previously diagnosed hypertension. The pre-intervention data for these subjects were published from the LIPGENE dietary intervention cohort elsewhere [29], [30]. Anthropometric measurements were recorded according to a standardized protocol for the LIPGENE study and blood pressure was measured according to the guidelines of European Society of Hypertension [31]. The present analyses include the 379 subjects with available SNP data. Of these participants, 68% received any kind of anti-hypertensive medication. Inclusion and exclusion criteria of the LIPGENE Study are presented in **table S1**.

### Biochemical measurements

Plasma, serum, and buffy coat were prepared from 12 h fasting blood samples in each subject. Serum insulin and C-peptide were measured by solid-phase, two-site fluoroimmunometric assay on a 1235 automatic immunoassay system (AutoDELFIA kits, Wallac Oy, Turku, Finland). Plasma glucose concentrations were measured using the IL TestTM Glucose Hexokinase Clinical Chemistry kit (Instrumentation Laboratories, Warrington, UK). Homeostasis model assessment of insulin resistance (HOMA-IR) was derived from fasting glucose and insulin levels [(fasting plasma glucose×fasting serum insulin)/22.5] [32]. As HOMA-IR takes into account both insulin and glucose levels, it may be a more complete index than plasma insulin. Homeostasis model assessment of β-cell function (HOMA-B) was calculated as [(20×fasting serum insulin)/(fasting plasma glucose−3.5)]. Cholesterol and triglycerides were quantified using the IL Test™ Cholesterol kit and IL Test™ Triglycerides kit (Instrumentation Laboratories, Warrington, UK).

Plasma fatty acid composition was determined as a biomarker of habitual dietary fat intake and reflects the combination of dietary fat consumption and endogenous de novo fatty acid biosynthesis and metabolism. Fatty acids were extracted from plasma and transmethylated with boron trifluoride in methanol. Fatty acid methyl esters were analyzed by gas chromatography on a Shimadzu GC-14A (Shimadzu, Kyoto, Japan) fitted with a Shimadzu C-r6A integrator and a 25M BP 21 polar aluminium silica column. Detector and injector temperatures were 260°C and 250°C respectively. Fatty acids were identified by the comparison of the relative retention times of plasma fatty acid methyl esters with fatty acid methyl esters standards. Fatty acid mass was measured as a relative percentage of the total quantified fatty acids [33]. Total plasma n-3 PUFA was calculated from the sum of C18:3 (n-3), C18:4(n-3), C20:4 (n-3), C20:5 (n-3), C22:5 (n-3) and C22:6 (n-3). Longchain (LC) n-3 PUFA was calculated from C20:5 (n-3) and C22:6(n-3); and n–6 PUFAs from the sum of 18:2, 18:3n–6, 20:3, 20:4n–6, and 22:4. Plasma concentrations of CRP were determined by high-sensitivity ELISA (BioCheck, Inc., Foster City, CA, USA) at the University College Dublin. Adiponectin was determined by use of ELISA (DuoSet® ELISA Development System, R&D Systems, MN, USA).

### SNP Selection and Genotyping

The rs1260326-P446L polymorphism was genotyped at the *GCKR* gene. DNA was extracted from buffy coat samples using the AutoPure LS automated system (Gentra Systems Inc., Minneapolis, MN, USA), and low yielding samples (<10 ng) were subjected to whole genome amplification using the REPLI-g kit (Qiagen Ltd. West Sussex, UK). Genotyping was conducted by Progenika Biopharma SA (Derio, Spain). Adherence to Hardy-Weinberg equilibrium (HWE) at each SNP locus was determined using the χ2 test with 1 degree of freedom.

### Statistical analysis

Biochemical variables were assessed for normality of distribution, and skewed variables were normalised by log10 or square root transformation as appropriate. Statistical analyses were carried out using SPSS version 18.0 for Windows (SPSS Inc, Chicago, IL). Data are presented as means ± standard error for continuous variables and as frequencies or percentages for categorical variables. Comparisons of frequencies between qualitative variables were carried out using the Chi-squared test. Potential confounding factors were age, sex, BMI, anti-hypertension pharmacological treatment and LIPGENE centre of origin. ANOVA-based models were used to test for associations between individual SNP and the variables studied, with the SNP as fixed factor, and age, BMI, sex and centre of origin as covariates. The effect of each SNP interacting with groups of plasma fatty acids (omega-3 and omega-6 polyunsaturated, saturated, monounsaturated) on each biochemical variable was investigated using the median of plasma fatty acids to dichotomize the population, and using the resulting groups (above-the-median versus below-the-median) as a fixed factor in combination with the SNP genotypes in an univariate ANOVA analysis with the same covariates and Bonferroni corrections as exposed above. Thus, in this model we could assess the associated effects of SNP alone, fatty acids alone and the interaction between the SNP and the fatty acids on the selected variables. Bonferroni's test was used in all cases where post-hoc was necessary.

## Supporting Information

Table S1 Inclusion and exclusion criteria of the LIPGENE Study.
(DOC)
