# Supplementary material for: Glucokinase Regulatory Protein Genetic Variant Interacts with Omega-3 PUFA to Influence Insulin Resistance and Inflammation in Metabolic Syndrome
Source: PLoS One. 2011 Jun 6;6(6):e20555. doi: 10.1371/journal.pone.0020555 (PMC3108949; doi:10.1371/journal.pone.0020555)
Supplement: Table S1 — Inclusion and exclusion criteria of the LIPGENE Study. (DOC) [file pone.0020555.s001.doc]

**Table S1: Inclusion and exclusion criteria of the LIPGENE Study.**

| **Inclusion Criteria**   - Age: 35 -70 years - Gender: males and females (not pregnant or lactating). - Body Mass Index (BMI) 20-40 kg/m^2^ - Total cholesterol concentration equal to or < 8.0 mmol/l. - Medications / nutritional supplements allowed, on condition that the subjects adhere to the same regimen during the intervention: anti-hypertensive medication (including beta-blockers), oral contraceptives, hormone replacement therapy, multi-vitamin supplements, and other non-fatty acid based nutritional supplements (e.g. garlic, anti-oxidants, etc). - Smokers and non-smokers. - Regular consumers of alcohol, which is not excessive as defined by elevated liver enzymes (AST and ALT). - Ethnicity: Intention to include white Europeans. |
| --- |
| **Exclusion Criteria**   - Age: < 35 or >70 years - Diabetes or other endocrine disorders. - Chronic inflammatory conditions. - Kidney or liver dysfunction. - Iron deficiency anaemia (haemoglobin < 12g/dl men, < 11g/dl women) - Prescribed hypolipidaemic medication - Prescribed anti-inflammatory medication - Fatty acid supplements including fish oils, evening primrose oil, etc. - Consumers of high doses of antioxidant vitamins (A, C, E, β-carotene). - Red rice yeast (Monascus purpureus) supplement usage. - High consumers of oily fish (> 2 serving of oily fish per week of herring, mackerel, kippers, pilchards, sardines, salmon, trout, tuna (fresh), crabmeat or marlin). One portion is defined as a small herring or mackerel, one can of salmon or sardines or one salmon or tuna steak. Tinned tuna is permitted as it contains only minor amounts of long chain n-3 PUFAs. - Highly trained or endurance athletes or those who participate in more than 3 periods of intense exercise per week. - Volunteers planning to start a special diet or lose weight (e.g. the Slimfast Plan, Atkins Diet etc). - Weight change equal or >3kg within the last 3 months. - Alcohol or drug abuse (based on clinical judgement). - Pregnant / lactating females / women planning a pregnancy in the next 12 months. Women who become pregnant during the dietary intervention period should be removed from the study. |
